# Polyphyllin I Inhibits the Metastasis of Cervical Cancer Through the Regulation of the β-Catenin Signaling Pathway

**DOI:** 10.3390/ijms26104630

**Published:** 2025-05-12

**Authors:** Yingbin Chai, Shaopeng Yu, Guoqiang Lin, Chunying Luo, Xu Wang, Rui Zhang, Jiawen Peng, Yuying Zhu, Jiange Zhang

**Affiliations:** 1 The Research Center of Chiral Drugs, Innovation Research Institute of Traditional Chinese Medicine, Shanghai University of Traditional Chinese Medicine, Shanghai 201203, China; chaiyb124@163.com (Y.C.); yushaopeng0405@163.com (S.Y.); lingq@sioc.ac.cn (G.L.); 13033721361@163.com (C.L.); rui_zhang@shutcm.edu.cn (R.Z.); pengjw1112@163.com (J.P.); 2 Institute of Chinese Materia Medica, Shanghai University of Traditional Chinese Medicine, Shanghai 201203, China; wangxucpu@163.com

**Keywords:** polyphyllin I, cervical cancer, metastasis, β-catenin, autophagy

## Abstract

Cervical cancer ranks as the fourth most prevalent cancer and cause of cancer-related mortality among women globally. It exhibits a recurrence/metastasis rate of approximately 30% and a dismal 5-year survival of only 17% in metastatic cases. Despite significant advancements in surgical techniques, chemoradiotherapy, and targeted therapies, effective treatment options for metastatic cervical cancer remain limited. This study explored Polyphyllin I (PPI), which is a monomeric compound derived from the Rhizoma of Paris Polyphyllin, as a potential inhibitor of cervical cancer metastasis. Mechanistically, PPI directly interacted with β-catenin at the Ser552 site, inhibiting its phosphorylation and subsequent nuclear translocation, thereby suppressing TCF/LEF transcriptional activity and downstream EMT transcription factors (ZEB1, Slug, Snail, and Twist). Notably, PPI promoted β-catenin degradation via the autophagy–lysosomal pathway, as confirmed by CHX chase assays and the detection of the p62 and LC3 proteins, without altering the mRNA levels of β-catenin. In vitro experiments demonstrated that PPI effectively suppressed the migration and invasion of HO-8910PM cells by reversing the process of EMT. Additionally, PPI effectively inhibited TCF/LEF signaling, leading to a reduction in the transcription levels of EMT-associated transcription factors (EMT-TFs), which was mediated by the TCF/LEF family downstream of β-catenin. Furthermore, PPI exhibited inhibitory effects on proliferation, migration, and invasion in both HPV-positive (SiHa) and HPV-negative (C33A) cervical cancer cells. In vivo, PPI significantly suppressed peritoneal metastasis in a luciferase-labeled HO-8910PM xenograft mouse model. These findings reveal the dual role of PPI in blocking β-catenin signaling and inducing β-catenin depletion, thereby effectively restraining metastatic progression. This study underscores the potential of PPI as a promising therapeutic candidate for targeting cervical cancer metastasis through autophagy-mediated β-catenin regulation, offering a novel strategy to address current treatment limitations.

## 1. Introduction

Cervical cancer ranks as the fourth most frequently diagnosed cancer and represents the fourth leading cause of cancer-related mortality among women globally [1]. Despite substantial progress in the screening, detection, and treatment of cervical lesions, cervical cancer continues to pose a significant global burden and remains a critical public health concern. In 2022, an estimated 661,021 new cases were reported, with 348,189 deaths being attributed to cervical cancer worldwide [2]. The metastasis of cervical cancer is a primary factor contributing to the high mortality rate associated with this disease. The 5-year survival rate for patients with recurrent or metastatic cervical cancer is merely 17% [3]. Pelvic lymph node metastasis represents a significant risk factor for cervical cancer recurrence, metastasis, and, ultimately, patient mortality [4].

In recent years, substantial advancements have been achieved in the management of cervical cancer, particularly in the refinement of therapeutic approaches and the implementation of innovative treatments. Surgical intervention continues to be the primary modality for patients with early-stage cervical cancer, especially in cases involving localized lesions. Procedures such as hysterectomy and radical hysterectomy are effective in treating early-stage cancer. However, patients with an advanced or metastatic disease cannot achieve a cure through surgical intervention alone, and these procedures may also have an impact on fertility [5]. For locally advanced or metastatic cervical cancer, radiotherapy and chemotherapy are commonly employed as standard treatment modalities. Radiotherapy, delivered via external beam radiation or brachytherapy, is capable of effectively treating tumors and controlling the progression of localized lesions. The integration of cisplatin-based chemotherapy with radiotherapy has been established as the standard-of-care regimen. Despite their efficacy in managing advanced cervical cancer, these treatments are associated with notable side effects, including immunosuppression and dermatological damage. Moreover, their therapeutic efficacy remains constrained in cases exhibiting drug resistance [6]. In recent years, immunotherapy and targeted therapy have emerged as innovative treatment modalities. PD-1 inhibitors and antibody–drug conjugates (e.g., tisotumab vedotin) have demonstrated significant promise in the management of advanced cervical cancer, particularly with regard to enhancing patient survival and quality of life. Nevertheless, these therapies are still in the optimization and investigation stages, and not all patients will benefit from them [7]. Additionally, the strategies for combination therapies are continuously evolving, particularly the integration of immunotherapy with conventional chemotherapy and radiotherapy. Studies have shown that these combination approaches can substantially enhance survival rates in patients with locally advanced cervical cancer; however, managing side effects and addressing treatment complexity continue to pose significant challenges [6]. In summary, the treatment of cervical cancer is advancing toward personalization and diversification. When determining an appropriate treatment plan, it is essential to comprehensively evaluate factors such as the stage of cancer, the patient’s overall health status, and specific fertility requirements.

A comparative analysis of gene expression profiles between 20 lymph node-negative and 19 lymph node-positive cervical cancer patients, utilizing a comprehensive set of 285 pathway signatures, highlighted the pivotal role of the β-catenin pathway in pelvic lymph node metastasis during the early stages of cervical cancer [8]. Wang et al. revealed that avenanthramide 2P and triptolide inhibit cervical carcinogenesis through two mechanisms—promoting β-catenin degradation and blocking its nuclear accumulation [9]. Extensive evidence has now confirmed that the development of cervical cancer is attributed to an imbalance in the dual functions of β-catenin, which are involved in structural maintenance and signal transduction [10].

β-catenin is the key mediator of the canonical Wnt signaling pathway. β-catenin signaling is activated in the presence of Wnt ligands. At the cell surface, the binding of Wnt ligands induces the heterodimerization of the frizzled (FZD) and LRP5/6 receptors, triggering conformational changes [11]. Subsequently, dishevelled (DVL) is recruited to the membrane through its interaction with the cytoplasmic domain of FZD [12]. DVL binds AXIN and facilitates the recruitment of the destruction complex to the membrane. Following the phosphorylation of the LRP5/6 cytoplasmic domain by kinases such as CDK14 and GSK3β, the association between the destruction complex and the membrane is further strengthened [13]. Consequently, the activity of the destruction complex in promoting β-catenin phosphorylation and degradation is inhibited. Unphosphorylated cytoplasmic β-catenin accumulates and translocates into the nucleus. As β-catenin lacks a DNA-binding domain, it activates transcription by binding to TCF/LEF family members, histone modifiers such as CREB-binding protein (CBP), and other transcription factors [13]. Upon nuclear translocation, β-catenin displaces the transcriptional repressor Groucho, which forms a complex with TCF/LEF members in the absence of Wnt signaling. The active β-catenin/TCF complex then initiates the activation of epithelial–mesenchymal transition-inducing–inducing transcription factor (EMT-TFs) genes that govern epithelial–mesenchymal transition (EMT) [14].

The process of EMT entails the alteration of epithelial cells into mesenchymal phenotypes through specific molecular mechanisms. Studies confirm that EMT-positive primary cervical carcinomas are clinically characterized by rapid progression, aggressive invasion, elevated metastatic capacity, and the loss of epithelial cohesion [15]. EMT also plays a crucial role in cervical cancer metastasis. During EMT, the zinc finger family of transcription factors, including SNAI1, Slug, Twist, and matrix metalloproteinases (MMPs), are upregulated, which correlates with enhanced tumor cell invasion and motility. ZEB1, another critical EMT transcription factor, demonstrates ectopic overexpression in endometrial cancer that correlates with poor differentiation, aggressive invasiveness, metastatic progression, and adverse clinical outcomes [16]. EMT markers such as SNAI1 and ZEB1 may serve as novel predictors of pelvic lymph node metastasis, making them promising therapeutic targets for cervical cancer patients [17].

Traditional Chinese medicines (TCMs) like Rhizoma Paridis, toad venom, Prunella vulgaris, and Solanum nigrum are increasingly utilized in anticancer therapy [18]. Rhizoma Paridis refers to the dried root and Rhizome of Paris Polyphlla, a perennial herbaceous plant that belongs to the Liliaceae family (according to the Chinese Pharmacopeia), or Melanthiaceae family (as per The World Flora Online). This plant is predominantly distributed across southwest China. Rhizoma Paridis is included in the Pharmacopoeia of the People’s Republic of China as a key TCM for its heat-clearing and detoxifying properties (Commission, 2015). In recent years, Rhizoma Paridis has gained recognition for its potent anti-tumor effects and has been extensively utilized in TCM prescriptions for cancer treatments. Notably, Louliang capsules and Ruanjian oral solution are among the commercially available Chinese patent medicines that prominently feature Rhizoma Paridis as their main ingredient. The saponins present in Rhizoma Paridis are the primary bioactive constituents, and their total content is designated as the quality control index components according to the Chinese Pharmacopoeia. Among these, Polyphyllin I (PPI) has undergone extensive research and shows a comprehensive array of pharmacological effects. PPI has been extensively documented as one of the primary bioactive constituents possessing anticancer properties [19]. The anti-tumor mechanism of PPI has been investigated in non-small-cell lung cancer [20], gastric cancer [21], colon cancer [22], and other malignancies. While existing studies have confirmed that PPI inhibits the proliferation of cervical cancer HeLa cells [23] and enhances cisplatin sensitivity in cisplatin-resistant cervical cancer cells [24], its potential role in other cervical cancer cell lines and cervical cancer metastasis remains largely unexplored. Consequently, it is crucial to investigate the impact of PPI on cervical cancer metastasis and to elucidate the underlying mechanisms.

Studies have demonstrated that PPI exhibits inhibitory effects on a wide range of cancer cells and is involved in various mechanisms. PPI triggers the AKT/GSK-3β-mediated ubiquitin proteasomal degradation pathway of β-catenin, subsequently attenuating the pro-oncogenic effect of liver cancer stem cells [25]. PPI was observed to decrease the mRNA and protein expression of surviving by inhibiting the Wnt pathway and preventing the nuclear translocation of β-catenin, thereby inducing apoptosis in the U266 and RPMI8226 multiple myeloma cells [26]. The inhibition of osteosarcoma in vitro and in vivo as a result of PPI therapy is achieved through the suppression of Wnt/β-catenin pathway activity [27]. The restraining effect and mechanism of PPI on various tumors have been extensively studied, but the potential relationship between PPI and the Wnt/β-catenin signaling pathway in cervical cancer metastasis is yet to be explored.

The objective of this study is to explore the inhibitory effects of PPI on cervical cancer tumor growth and metastasis, as well as its interaction with β-catenin, with the aim of elucidating the underlying mechanism by which PPI influences cervical cancer metastasis. This study may provide new insights and directions for the development of novel anti-metastatic drugs targeting cervical cancer.

## 2. Results

### 2.1. The Interaction Between PPI and β-Catenin

**Figure 1:**
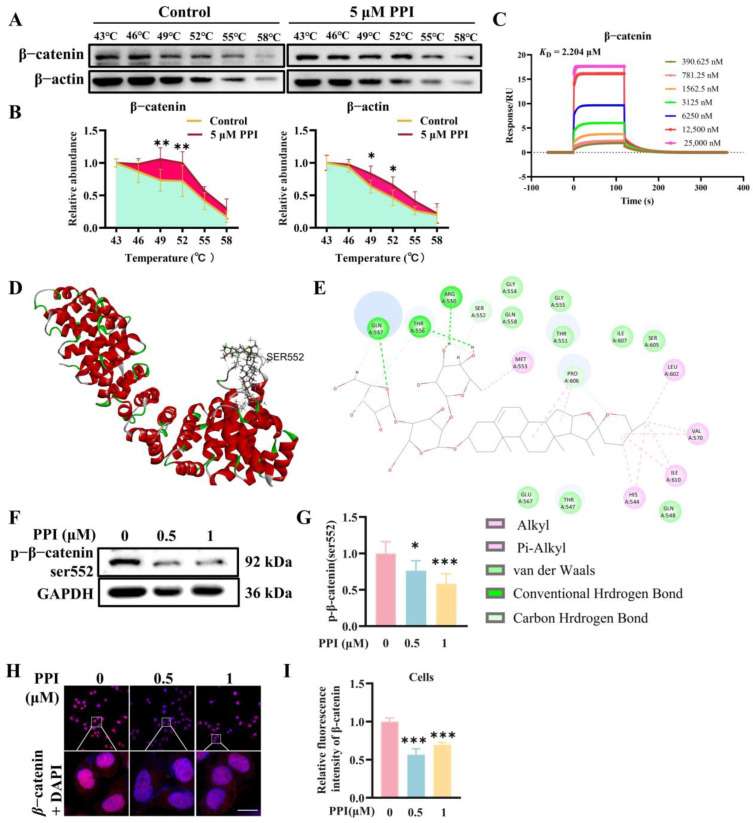
The interaction between PPI and β-catenin. (**A**,**B**) The thermostability of PPI on the β-catenin protein was confirmed through CETSA. (**C**) SPR assay for the identification of PPI targeting the β-catenin protein. (**D**) Spatial structure modeling of PPI and the serine 552 site of β-catenin. Polyphyllin I docked into the cavity. (**E**) The binding mode of Polyphyllin I and β-catenin. (**F**,**G**) A Western blot assay was performed to assess the impact of various concentrations of PPI on the alterations in p-β-catenin (ser552) protein levels after a 6-h treatment of HO-8910PM cells, followed by quantitative data analysis. (**H**,**I**) An immunofluorescence assay was conducted to detect the effect of different concentrations of PPI on the changes in the nucleoplasmic distribution of β-catenin protein after a 6-h treatment of HO-8910PM cells. Data from six independent experiments are presented as mean ± S.D. *: *p* < 0.05; **: *p* < 0.01; ***: *p* < 0.001 vs. control.

The interaction between PPI and β-catenin protein was investigated by conducting a cellular thermal shift assay (CETSA). PPI significantly enhanced the thermal stability of β-catenin within the temperature range of 43 to 58 °C (Figure 1A,B), which represented the binding capacity of PPI to β-catenin protein in cells. Surface plasmon resonance (SPR) experiments demonstrated a robust binding affinity between PPI and β-catenin, with a K_D_ value of 2.204 μmol/L (Figure 1C). To enhance the understanding of the interaction between PPI and β-catenin protein, we subsequently employed computational docking to simulate the binding patterns. As can be seen in Figure 1D,E, the ligand extended deeply into the cavity beside the side chain containing Ser552, which resulted in a conformational change to the side chain. Additionally, PPI has a carbon–hydrogen interaction with Ser552. Simultaneously, it can be observed that the molecule reports hydrogen bond interactions with Arg550, Thr556, and Gln557. Some weak alkyl interactions were also identified. According to the docking results, the binding of PPI to β-catenin protein may impact the function of Ser552, whose phosphorylation could modulate the stability of β-catenin. Thus, we assessed the phosphorylation of Ser552 in β-catenin protein. As shown in Figure 1F,G, the levels of phosphorylated β-catenin (Ser552) in HO-8910PM cells were significantly reduced following PPI treatment.

Furthermore, the immunofluorescence staining results demonstrated a significant reduction in β-catenin expression in HO-8910PM cells treated with 0.5 μM and 1 μM PPI (Figure 1H,I). The experimental results demonstrate that PPI effectively downregulates the expression of total β-catenin, both in the cytoplasm and nucleus. The phosphorylation of β-catenin at Ser552 by AKT has been documented to induce dissociation from intercellular contacts and subsequent nuclear accumulation [28]. The reduction in nuclear β-catenin expression induced by PPI treatment was consistent with the decrease in phosphorylated β-catenin (Ser552). This additional finding prompted us to investigate the mechanism by which PPI degrades β-catenin.

### 2.2. The Depletion of β-Catenin Triggered by PPI Is Mediated Through the Process of Autophagy

**Figure 2:**
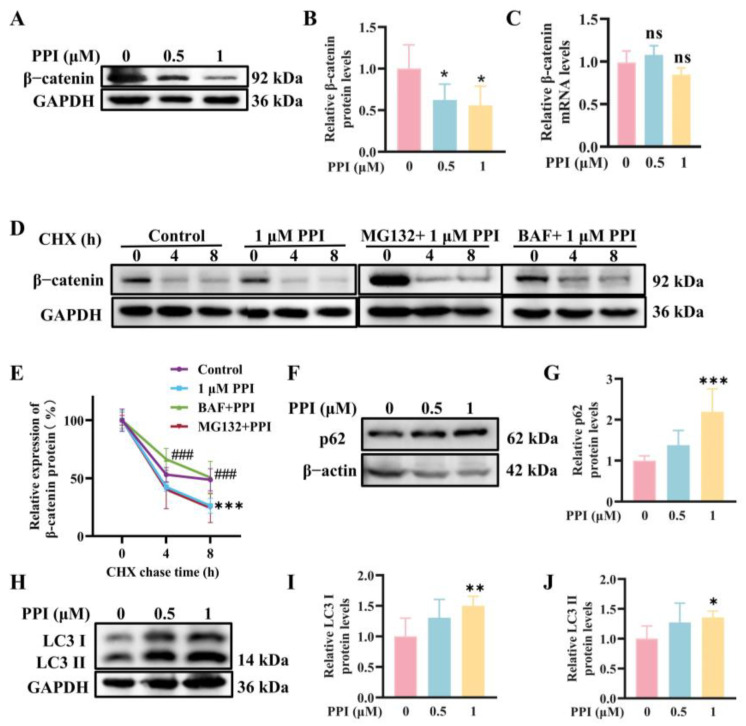
The depletion of β-catenin triggered by PPI is mediated through the process of autophagy. (**A**,**B**) The effect of different concentrations of PPI on the expression levels of β-catenin protein in HO-8910PM cells following a 6-h treatment was assessed using a Western blot assay, and the resulting data were further subjected to quantitative analysis. (**C**) qRT-PCR analysis of β-catenin mRNA levels in PPI-treated cells. (**D**) Assessment of the impact of PPI on β-catenin protein degradation: HO-8910PM cells were exposed to PPI (1 μM) either alone or in combination with either MG132 (20 μM), which is a proteasome inhibitor, or BAF (200 nM), which is an autophagy inhibitor, for 6 h. Subsequently, the cells were subjected to CHX (100 µg/mL) treatment for the indicated durations. (**E**) The quantification results of (**D**). (**F**,**G**) The effect of different concentrations of PPI on the expression levels of p62 protein in HO-8910PM cells following a 6-h treatment was assessed by WB, and the resulting data were subjected to quantitative analysis. (**H**–**J**) The effect of different concentrations of PPI on the expression levels of LC3 I and LC3 II proteins in HO-8910PM cells following a 6-h treatment was assessed by WB, and the resulting data were subjected to quantitative analysis. Data from six independent experiments are presented as mean ± S.D. *: *p* < 0.05; **: *p* < 0.01; ***: *p* < 0.001 vs. control; ###: *p* < 0.001 vs. 1 μM PPI.

We further explored the intrinsic mechanism underlying the PPI regulation of β-catenin. Previous experiments have confirmed that PPI downregulates the protein expression of β-catenin (Figure 2A,B), while no significant downregulation was observed in the mRNA expression levels of β-catenin by PPI (Figure 2C). In the presence of protein synthesis inhibitor cycloheximide (CHX), HO-8910PM cells exhibited an accelerated degradation of β-catenin mediated by PPI, which consequently reduced the half-life (t_1/2_) of β-catenin from 7.5 ± 1.0 h (control condition) to 3.2 ± 0.3 h. Additionally, the degradation of β-catenin induced by PPI can be reversed upon treatment with the autophagy inhibitor bafilomycin (BAF), with a half-life (t_1/2_) of 4.9 ± 0.5 h. In contrast, treatment with the proteasome inhibitor MG132 exhibits a minimal effect on β-catenin degradation, with a half-life (t_1/2_) of 2.4 ± 0.2 h. The data presented herein demonstrate that PPI effectively inhibits β-catenin degradation through the autophagy–lysosome pathway.

To elucidate the potential mechanisms underlying the reduction in β-catenin protein levels, the expression levels of p62/SQSTM1 and LC3B proteins were detected using WB analysis. A 6-h treatment with PPI significantly upregulated the protein levels of p62 (Figure 2F,G), as well as of LC3 I and LC3 II, in HO-8910PM cells (Figure 2H–J).

A significant increase in LC3 II protein expression was observed, providing evidence that autophagy is activated. Additionally, the increased expression of p62 demonstrates that autophagy is induced by PPI through the phosphorylation of the Ser552 site of β-catenin. PPI may upregulate LC3 expression at the transcriptional level by stabilizing LC3 mRNA, thereby increasing the pool of LC3 I available for lipidation and conversion to LC3 II.

The regulatory feedback mechanism between Wnt/β-catenin signaling and autophagy has been extensively characterized at multiple levels. The Wnt/β-catenin pathway exerts a vital role in negatively regulating autophagy and suppressing p62/SQSTM1 expression [29]. The activation of the Wnt/β-catenin signaling pathway leads to the stabilization and subsequent nuclear accumulation of β-catenin. This accumulated β-catenin subsequently translocates into the nucleus, where it interacts with TCF/LEF family members to modulate the transcription of target genes [30]. Furthermore, the inhibition of β-catenin and TCF signaling has been demonstrated to upregulate p62/SQSTM1 expression, thereby activating the autophagy machinery [31]. Additionally, the phosphorylation of β-catenin at Ser 552 mediated by AKT increases TCF/LEF transcriptional activity, thus promoting tumor cell invasion [28].

### 2.3. PPI Regulates the Expression of EMT-TFs by Modulating the β-Catenin Signaling Pathway

**Figure 3:**
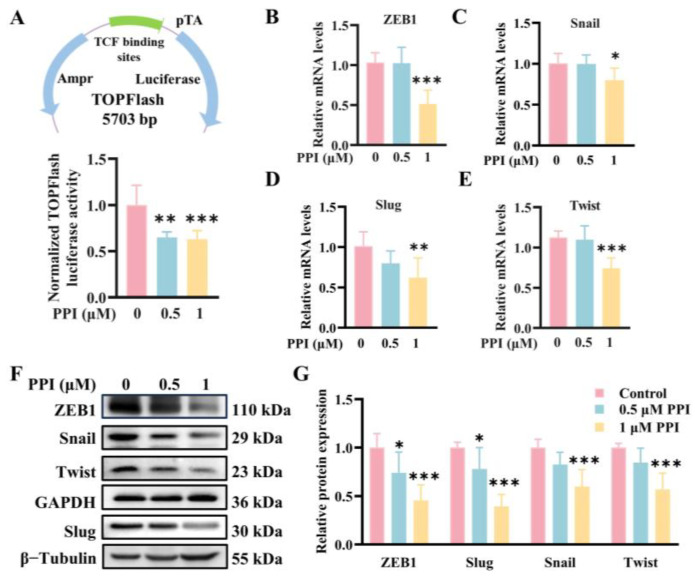
PPI regulates the expression of EMT-TFs by modulating the β-catenin signaling pathway. (**A**) TOPFlash assays were performed on HO-8910PM cells treated with PPI at the indicated concentrations for 6 h. The presented data are normalized to the activities of Renilla luciferase from six independent experiments. (**B**–**E**) qRT-PCR analysis of the mRNA levels of ZEB1, Snail, Slug, and Twist in HO-8910PM cells treated with PPI. (**F**,**G**) The protein expression levels of ZEB1, Slug, Snail, and Twist in HO-8910PM cells were assessed using a Western Blot assay, followed by quantitative data analysis. Data from six independent experiments are presented as mean ± S.D. *: *p* < 0.05; **: *p* < 0.01; ***: *p* < 0.001 vs. control.

A significant inhibition of Wnt signaling by PPI was confirmed using a TOPFlash (TCF/LEF reporter) luciferase assay (Figure 3A). The downregulation of ZEB1, Twist, Slug, and Snail mRNA levels (Figure 3B–E) and the reduction in protein levels (Figure 3F,G) in HO-8910PM cells after PPI treatment were further confirmed through quantitative RT-PCR and Western Blot experiments. Importantly, a dose-responsive decrease in these protein levels was detected following PPI treatment. The findings suggest that the dampening effect of PPI on cervical cancer metastasis may be mediated via the modulation of the β-catenin signaling pathway.

### 2.4. PPI Inhibits the Growth, Migration, and Invasion of HO-8910PM Cells by Reversing the EMT Progress

**Figure 4:**
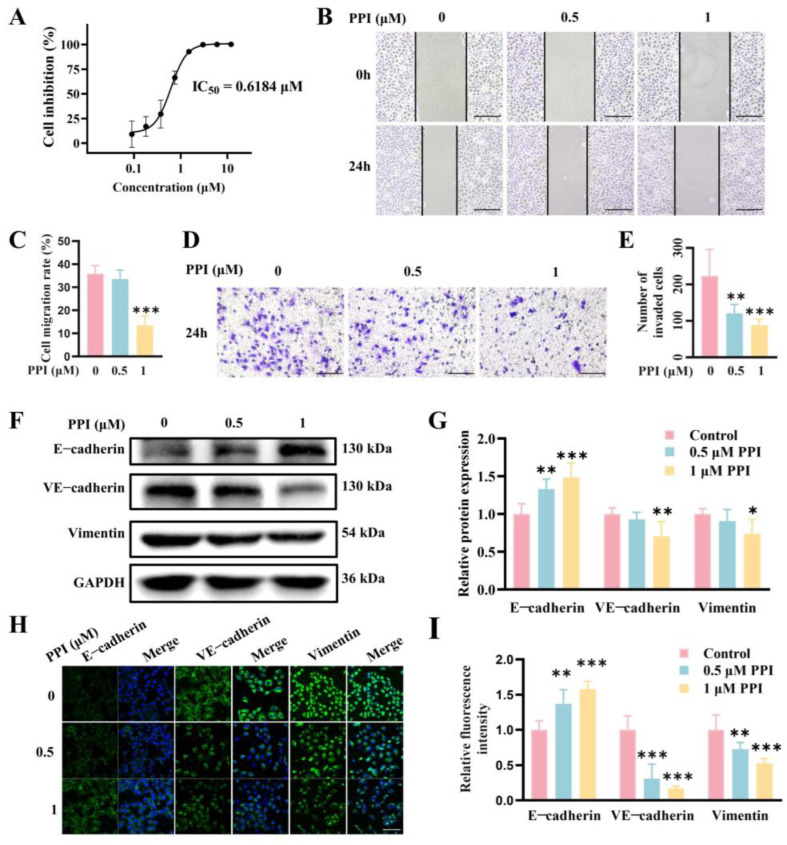
PPI inhibits the growth, migration, and invasion of HO-8910PM cells by reversing the EMT progress. (**A**) Cell proliferation was evaluated at 48 h using the MTT assay, and the IC_50_ values were determined from dose–response curves generated with GraphPad Prism 8. (**B**) The wound-healing assay was conducted in HO-8910PM cells to evaluate cell migration. Images were captured at 0 and 24 h. The scale is 500 μm. (**C**) The data quantification results of (**B**). (**D**) A transwell migration assay was performed to monitor the rate of cellular migration. The scale is 500 μm. (**E**) The quantification results of (**D**). (**F**) The protein expression levels of E-cadherin, VE-cadherin, and Vimentin in HO-8910PM cells. (**G**) The quantification results of (**F**). (**H**) An immunofluorescence assay was performed to detect E-cadherin, VE-cadherin, and Vimentin expression in HO-8910PM cells treated with PPI. The scale is 100 μm. (**I**) The quantification results of (**H**). Data from six independent experiments are presented as mean ± S.D. *: *p* < 0.05; **: *p* < 0.01; ***: *p* < 0.001 vs. control.

The anticancer effect of PPI on HO-8910PM cells was initially assessed in this study using the MTT assay to determine cell viability. The findings demonstrated that PPI effectively suppressed cell proliferation, exhibiting a dose-dependent inhibitory effect with an IC_50_ value of 0.6184 μM at 48 h (Figure 4A). The wound-healing assay is a straightforward and intuitive method for evaluating cell migration capacity. HO-8910PM cells treated with 1 μM PPI displayed markedly divergent abilities in terms of wound healing (Figure 4B,C); the migration rate decreased significantly from 40% to 10%~20%. To further investigate the effects of PPI, we conducted a transwell assay to gauge cell invasion at 0, 0.5, and 1 μM PPI; the invasion of HO-8910PM cells observed a marked drop with the increasing dose (Figure 4D,E). These observations demonstrated that PPI optimally inhibits the migration and invasion of HO-8910PM cells in a dose-dependent manner.

The activation of epithelial–mesenchymal transition (EMT) is a crucial mechanism in the course of cancer cell metastasis, wherein epithelial cells adopt mesenchymal cell traits, including boosted cell motility and migration. EMT is characterized by the loss of epithelial cell markers such as E-cadherin, as well as an elevated level of mesenchymal cell markers like VE-cadherin and Vimentin. The protein expression levels of E-cadherin, VE-cadherin, and Vimentin were assessed in HO-8910PM cells using Western blot detection. The expression of E-cadherin was significantly increased when treated with 0.5 μM and 1 μM PPI, while VE-cadherin and Vimentin exhibited a significant decrease when treated with 1 μM PPI (Figure 4F,G). Meanwhile, the immunofluorescence assay revealed a significant increase in E-cadherin staining and a significant decrease in VE-cadherin and Vimentin staining in the PPI-treated groups (0.5 μM and 1 μM PPI) compared to the control group (Figure 4H,I).

### 2.5. PPI Inhibits the Proliferation, Migration, and Invasion of Cervical Cancer Cell Lines SiHa and C33A

**Figure 5:**
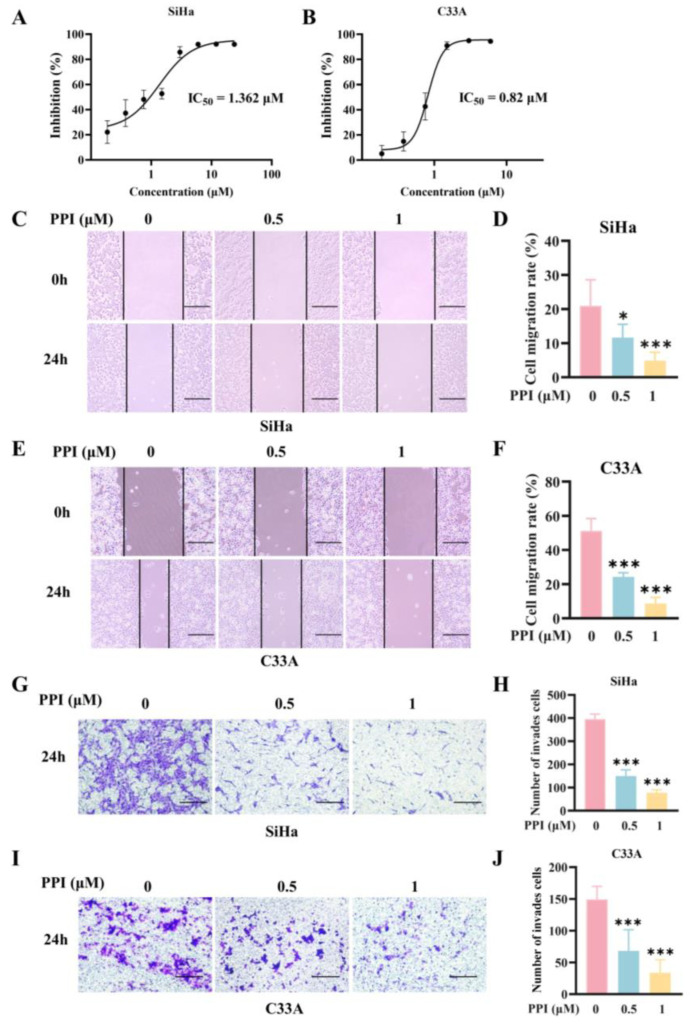
PPI inhibits the proliferation, migration, and invasion of cervical cancer cell lines SiHa and C33A. (**A**,**B**) Dose–response curves of PPI-treated (**A**) SiHa and (**B**) C33A cells after 48 h, quantified using the MTT assay. The IC_50_ values were calculated by utilizing GraphPad Prism 8 software for analysis. (**C**–**F**) The wound-healing assay demonstrated the migration capability of SiHa (**C**,**D**) and C33A (**E**,**F**) cells following treatment with PPI. Representative images ((**C**,**E**) scale bar = 50 μm) and quantitative analysis (**D**,**F**) at 0 and 24 h are presented. (**G**–**J**) The transwell migration assay revealed the PPI-mediated suppression of SiHa (**G**,**H**) and C33A (**I**,**J**) cell invasion. Representative images ((**G**,**I**) scale bar = 500 μm) and quantification (**H**,**J**) at concentrations of 0, 0.5, and 1 μM PPI are presented. Data are derived from six independent experiments and are expressed as mean ± S.D. Statistical significance was determined relative to the control group—*: *p* < 0.05; ***: *p* < 0.001.

This study further assessed the inhibitory effect of PPI on SiHa and C33A cells by evaluating cell viability using the MTT assay. The findings revealed that PPI significantly suppressed the proliferation of both SiHa and C33A cells in a dose-dependent manner, with IC_50_ values of 1.362 μM and 0.82 μM at 48 h, respectively (Figure 5A,B). A wound-healing assay was performed to systematically evaluate cell migration, assessing the impact of PPI on the migration ability of SiHa and C33A cells. The results demonstrated that treatment with 0.5 μM and 1 μM PPI significantly impaired cell migration, leading to a reduced migration rate in SiHa cells (Figure 5C,D) and C33A cells (Figure 5E,F). To further investigate the effects of PPI, transwell invasion assays were performed to comprehensively assess the invasion capability of SiHa and C33A cells at concentrations of 0, 0.5, and 1 μM PPI. The results demonstrated that with the increase in PPI concentration, the invasion capability of both SiHa and C33A cells was significantly decreased (Figure 5G–J). These observations suggest that PPI exhibits a significant inhibitory effect on the migration and invasion capabilities of SiHa and C33A cells in a dose-dependent manner.

### 2.6. PPI Inhibits the Growth of Cervical Cancer Cells In Vivo

**Figure 6:**
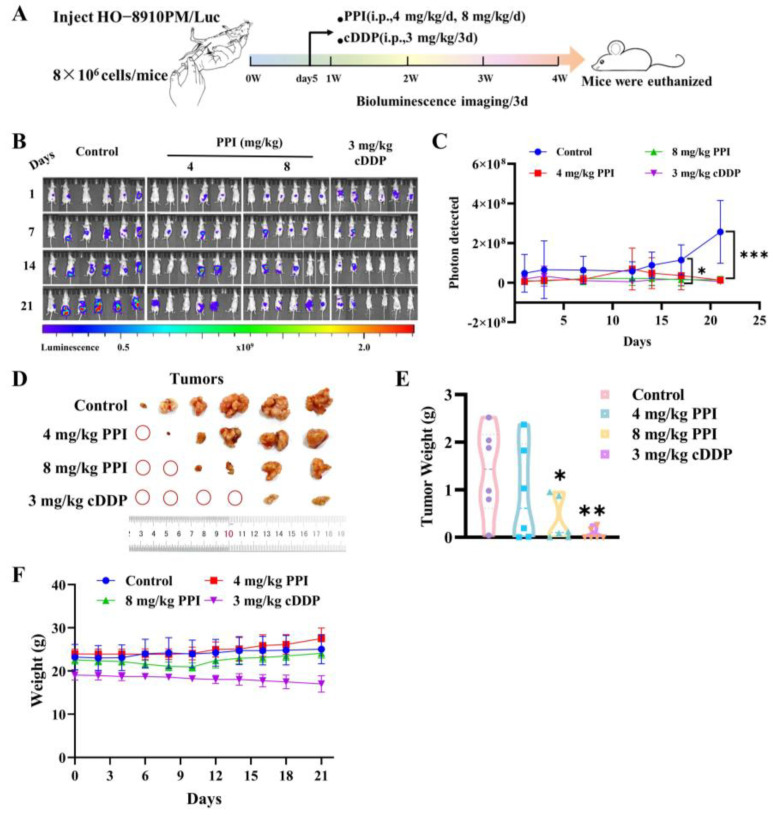
PPI inhibits the growth of cervical cancer cells in vivo. (**A**) A schematic depiction of therapeutic regimens for a peritoneal metastasis model of cervical cancer. (**B**,**C**) The images of distinct cohorts of HO-8910PM-luci-bearing mice (**B**), accompanied by quantitative statistical analysis of bioluminescence data (**C**). (**D**,**E**) Images illustrating abdominal tumors in nude mice from both the control group and the drug administration groups (**D**), as well as the statistical analysis of tumor weight in nude mice for both groups (**E**). (**F**) The dynamics of body weight were systematically evaluated over a 21-day treatment period across four experimental groups. Six animal samples were included in each group (*n* = 6), with data presented as mean ± S.D. Statistical significance was defined as follows: *—*p* < 0.05; **—*p* < 0.01; and ***—*p* < 0.001 compared to the control group.

The findings from in vitro studies have provided data demonstrating that PPI exhibits inhibitory effects regarding the migration and invasion of cervical cancer cells by reversing the process of EMT. Consequently, our research will proceed to give additional proof of the in vivo anti-metastatic potential of PPI on cervical cancer through the intraperitoneal injection of luciferase-labeled HO-8910PM cells in nude mice. Subsequently, once the level of bioluminescence reached 1 × 10^7^, tumor-bearing mice were randomly assigned to one of four groups—control group, 4 mg/kg PPI group, 8 mg/kg PPI group, and 3 mg/kg cisplatin (cDDP) group. The PPI was delivered via intraperitoneal injection at the designated dose on a daily basis, while cisplatin was given via intraperitoneal injection at the prescribed dose once every four days. All treatments lasted for a duration of 3 weeks (Figure 6A). The bioluminescence signal of the mice in the control group exhibited a progressive increase over time, whereas tumor growth and spread were significantly suppressed in the mice receiving 4 or 8 mg/kg of PPI (Figure 6B,C). The mice in the control group exhibited a significant incidence and size of tumors within the peritoneal space and pectoral cavity when compared to the groups treated with PPI (Figure 6D,E). Throughout the 21-day treatment period, body weight changes (Figure 6F) were systematically monitored as an indicator of treatment tolerability. The results indicated that mice treated with 4 mg/kg and 8 mg/kg PPI exhibited no significant differences in body weight compared to the control group, and the 3 mg/kg cDDP-treated group demonstrated a mild reduction in body weight by day 21. These findings suggest that PPI maintains a favorable safety profile at the tested doses, whereas cisplatin administration led to observable toxicity, as evidenced by body weight loss.

### 2.7. PPI Inhibits the Metastasis of Cervical Cancer Cells In Vivo

**Figure 7:**
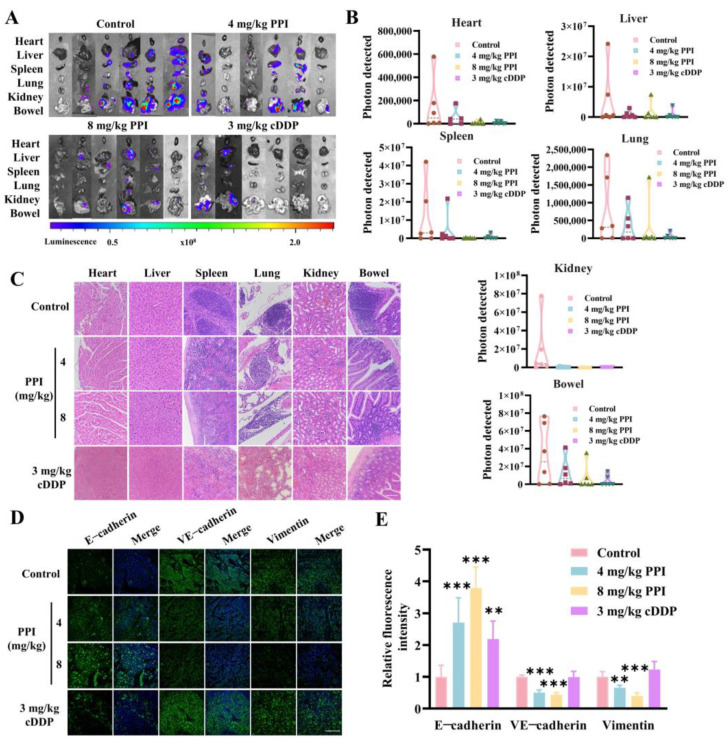
PPI inhibits the metastasis of cervical cancer cells in vivo. (**A**,**B**) Bioluminescence imaging of various organs exhibiting metastatic or disseminated cancer cells (**A**) and a quantitative analysis of total bioluminescence in each group of organs (**B**). (**C**) H&E staining of various organs in each group. The scale is 100 μm. (**D**,**E**) The immunofluorescence staining of E-cadherin, VE-cadherin, and Vimentin was performed, followed by data quantification analysis, comparing the control group with the treated group. Six animal samples were used per group (*n* = 6), and the results are presented as mean ± S.D. **: *p* < 0.01; ***: *p* < 0.001 vs. control. The scale is 100 μm.

In addition to the growth inhibition in cervical cancer tumors by PPI, we also discovered that PPI-treated mice exhibited reduced dissemination and fewer metastases in various organs (e.g., heart, liver, spleen, lung, kidney, and bowel) relative to the control group (Figure 7A). The fluorescence quantitative analysis of the total viscera from each group of six mice revealed a marked diminution in the total fluorescence intensity in the viscera of mice treated with 8 mg/kg PPI (Figure 7B). Subsequently, the viscera sections were prepared and stained with hematoxylin–eosin (HE). In the control group, spleen, lung, and bowel sections exhibited a higher density of blood vessels and an increased presence of metastatic foci, whereas such occurrences were infrequent in the PPI-treated groups. The histological examination of various organs in each group revealed a significant reduction in cervical cancer cell metastasis following the administration of PPI (Figure 7C). We then employed immunofluorescence to detect the expression of EMT marker proteins in tumor tissues. As opposed to the control group, there was a significant upregulation in the expression of the epithelial cell marker protein E-cadherin, whereas expressions of Vimentin and VE-cadherin were significantly decreased in the PPI treatment groups (Figure 7D,E). The above results further substantiate the restraining effect of PPI on cervical cancer metastasis by effectively reversing the process of EMT.

### 2.8. PPI Suppresses the Growth and Metastasis of Cervical Cancer Through the Modulation of the β-Catenin Signaling Pathway

**Figure 8:**
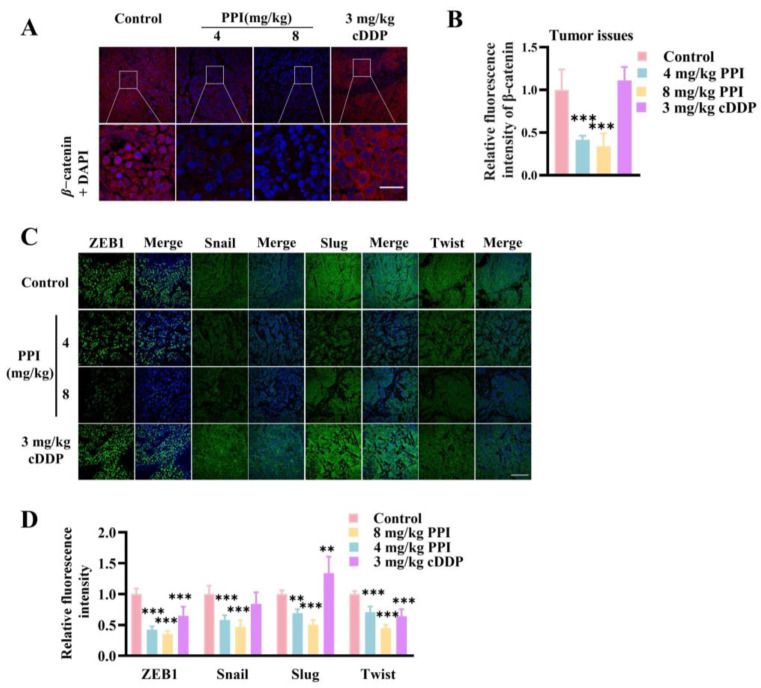
PPI suppresses the growth and metastasis of cervical cancer through the modulation of the β-catenin signaling pathway. (**A**,**B**) Immunofluorescence staining and quantification of β-catenin in cervical cancer tumor tissue sections. (**C**,**D**) Immunofluorescence staining and fluorescence analysis were performed on tumor paraffin sections to detect the expression of ZEB1, Snail, Slug, and Twist proteins. Six animal samples were used per group (*n* = 6), and the results are presented as mean ± S.D. **: *p* < 0.01; ***: *p* < 0.001 vs. control.

Many studies have demonstrated that targeting the β-catenin signaling pathway may confer therapeutic benefits in cervical cancer. β-catenin plays a pivotal role in initiating the transcription of target genes within the classical Wnt signaling pathway. Hence, we investigated the influence of PPI on the expression of β-catenin protein in HO-8910PM cell tumor tissue. Interestingly, the immunofluorescent staining of tumor paraffin sections revealed a significant downregulation of β-catenin protein expression in the PPI-treated group compared to the control groups (Figure 8A,B). The process of EMT involves transcriptional reprogramming, which is orchestrated by specific EMT transcription factors (EMT-TFs). Former studies have established that the transcription of EMT-TFs is mediated by the TCF/LEF family downstream of β-catenin. In an attempt to further illustrate the precise molecular interactions pertinent to the PPI inhibition of cervical cancer metastasis, an immunofluorescence assay was conducted to assess the expression levels and localization patterns of EMT-TFs. The fluorescence intensities of ZEB1, Twist, Slug, and Snail in the tumor tissues of the PPI treatment groups presented a notable decrease in comparison with those in the control group (Figure 8C,D). The findings suggest that the dampening effect of PPI on cervical cancer metastasis may be mediated via the modulation of the β-catenin signaling pathway.

## 3. Discussion

Cervical cancer represents one of the most prevalent malignancies among women globally. Owing to the advancements in precancerous screening techniques and the widespread implementation of human papillomavirus (HPV) vaccination programs, both the incidence and mortality rates associated with cervical cancer have declined in high-income countries. Nevertheless, in low- and middle-income countries, cervical cancer continues to impose a substantial health burden, significantly threatening women’s well-being, as reported by the authors of [32]. Consequently, further fundamental research into the molecular mechanisms underlying cervical cancer remains critically important.

Persistent HPV infection is uniquely essential for the progression of cervical cancer, setting it apart from other types of cancer. During the course of HPV infection, acting as a cancer initiator, three key events occur: the integration of viral DNA into the host genome, the expression of viral oncoproteins (E6 and E7) in epithelial cells, and the interaction between viral oncoproteins and cellular proteins [33]. Research indicates that the viral oncoproteins E6 and E7 are significantly overexpressed in cervical cancer tissues [34]. As a result, the expression of these oncoproteins in epithelial cells, as well as their interactions with host cellular proteins, has become a primary focus in studies of HPV-driven oncogenesis. β-catenin has been demonstrated to accelerate HPV-16-mediated cervical carcinogenesis in transgenic mice. Moreover, the activation of the Wnt/β-catenin signaling pathway is required for the transformation of HPV-expressing human keratinocytes [9,35,36,37]. Both E6 and E7 upregulate β-catenin expression and enhance TCF-mediated transcription. This effect is primarily attributed to the decreased levels of the E3 ubiquitin ligase Siah-1, which functions as a β-transducin repeat-containing protein (β-TrCP) substitute to promote β-catenin degradation [38] through a mechanism that is independent of the phosphorylation sites typically recognized by β-TrCP. In an immortalized human keratinocyte model transformed by the HPV oncoproteins E6/E7 and further modified with SV40 small T antigen (smt), Uren et al. discovered that smt directly inactivates PP2A, consequently leading to β-catenin accumulation [36]. Moreover, E7 strongly binds to the catalytic subunit of PP2A, thereby suppressing its activity and contributing to the stabilization of cytoplasmic β-catenin [39]. Based on previous evidence, HPV oncoproteins have been shown to interact directly or indirectly with β-catenin, subsequently activating β-catenin signaling and, ultimately, facilitating cervical carcinogenesis. Consequently, HPV may serve as the initiating factor in this multistep tumorigenesis process, while the alterations in β-catenin and the hyperactivation of the Wnt/β-catenin pathway in cervical cancer cells constitute the second factor required for full malignant progression.

The significant role of TCM in anti-tumor therapy has been demonstrated in previous studies, elucidating its diverse mechanisms and pathways. Utilizing TCM for the prevention of tumor recurrence not only exhibits a commendable therapeutic effect but also effectively ameliorates the adverse reactions associated with cancer treatment, thereby establishing a robust theoretical foundation for clinical management and medication. The Paridis Rhizoma is a natural herbal medicine that was initially documented in the Shennong Herbal Classic. In clinical settings, it is employed for the cure of dysfunctional uterine bleeding, neurodermatitis, surgical inflammation, and cancer, exhibiting remarkable therapeutic efficacy. The Paridis Rhizoma is a key ingredient in several renowned Chinese patent medicines with anti-tumor properties, including Loulian capsule and Gongxuening capsule. PPI is a prominent saponin component found in Rhizoma Paridis, which has been scientifically proven to effectively inhibit the metastasis of various types of tumor cells. For instance, it attenuates invasion and metastasis among drug-resistant hepatocellular carcinoma cells [40], as well as inhibiting invasion and epithelial-mesenchymal transition in prostate cancer [41]. While existing studies have confirmed that PPI inhibits the proliferation of cervical cancer HeLa cells [23] and enhances cisplatin sensitivity in cisplatin-resistant cervical cancer cells [24], its potential role in other cervical cancer cell lines and cervical cancer metastasis remains unexplored. Therefore, it is imperative to examine the impact of PPI on cervical cancer metastasis and elucidate its underlying mechanism.

In this study, we demonstrated direct binding between PPI and β-catenin protein, which was validated by the CETSA assay and the SPR assay. As Ser552 in β-catenin exhibits an essential role in many pharmacological processes, the amino acid residue has become an important point in the development of new therapeutics, especially in cancer research [42,43,44,45]. In this work, we applied flexible docking to discover the interaction mode between Polyphyllin I and β-catenin. From the docking results, we found that Polyphyllin I can be docked into the cavity of β-catenin and can directly bind to Ser552. This interaction prohibits other interactions with the amino acid residue. Simultaneously, the ligand obviously changed the conformation of the side chain, which also significantly influenced the interaction between β-catenin and other proteins. Notably, PPI also inhibits phosphorylation of β-catenin at Ser552. Furthermore, the administration of PPI resulted in a reduction in β-catenin protein expression, both in the nucleus and cytoplasm, while the mRNA levels of β-catenin remained unchanged. The CHX chase experiment, along with the increased expression of p62 and LC3 II, demonstrates that PPI triggers the degradation of β-catenin protein via the autophagy–lysosome pathway. Consequently, our findings provide evidence that PPI facilitates the autophagy-mediated degradation of β-catenin protein instead of affecting its mRNA expression level. We have confirmed that PPI effectively suppresses the migration and invasion of HO-8910PM cells by reversing the process of EMT, thereby inhibiting the growth and metastasis of cervical cancer cells in vivo. Notably, PPI exhibits anti-proliferative, anti-migratory, and anti-invasive effects on both HPV-infected cervical cancer cell line SiHa and HPV-negative cervical cancer cell line C33A in vitro. Furthermore, PPI downregulates β-catenin protein expression and TCF/LEF transcriptional activity, resulting in decreased levels of EMT-related transcription factors at both the mRNA and protein levels. Collectively, these results yield persuasive evidence for the role of PPI in suppressing cervical cancer metastasis through the modulation of the β-catenin signaling pathway, with a particular emphasis on targeting β-catenin.

The stabilization of β-catenin is crucial for cancer metastasis and is typically determined by the delicate balance between phosphorylation and dephosphorylation activities, which are regulated by various kinases and phosphatases. The phosphorylation of β-catenin at Ser552 enhances the transcriptional activity of TCF/LEF and promotes cancer invasion and cell EMT [28,45]. Through computational simulation, we identified that PPI could bind to Ser552 of β-catenin. In our work, PPI treatment led to a reduction in the protein levels of phosphorylated β-catenin (Ser552). While the study by Liao et al. [25] demonstrated that Polyphyllin I (PPI) can bind to AKT and the existing literature suggests that AKT can phosphorylate β-catenin at Ser552 [28], the data from Liao et al.’s study revealed no significant alteration in the total AKT expression following PPI treatment. Consequently, this indicates that the reduction in p-β-catenin (Ser552) induced by PPI is unlikely to be attributed to AKT degradation. Our findings also demonstrate that PPI treatment effectively suppressed the transcriptional activity of TCF/LEF, as well as the expression of EMT-related transcription factors such as Snail, Slug, Twist, and ZEB1. These observations provide mechanistic insights into the inhibitory effects of PPI on tumor metastasis.

The process of autophagy, which relies on lysosome functions and serves as a fundamental cellular degradation mechanism, performs crucial roles in sustaining cell homeostasis, modulating cell signaling pathways, and being intricately involved in various pathological conditions [46]. LC3 I and LC3 II are two key forms of the LC3 protein during autophagy, critically contributing to the formation and functionality of autophagosomes. LC3 I is the unmodified form of the protein, residing in the cytoplasm, whereas LC3 II represents the lipidated form, which accumulates on autophagosome membranes as autophagy progresses. The conversion of LC3 I to LC3 II constitutes a critical step in the initiation of autophagy and is dependent on the activity of autophagy-related proteins, such as ATG7 and ATG3. After lipidation, LC3 II specifically associates with the autophagosome membrane, thereby facilitating the expansion and maturation of autophagosomes while assisting in the encapsulation and degradation of cellular components, including damaged organelles and protein aggregates. The accumulation of LC3 II is frequently employed as an indicator of autophagy activation, with its levels exhibiting a positive correlation with autophagic flux. The dynamic interconversion between LC3 I and LC3 II plays a central role in sustaining the equilibrium of autophagic activity, especially in modulating the formation, maturation, and lysosomal fusion of autophagosomes. Changes in LC3 II levels are indicative of alterations in autophagic activity. Specifically, an increase in LC3 II generally corresponds to the upregulation of autophagy, whereas a decrease is often associated with the inhibition of the autophagy process [47]. In our study, a significant increase in the expression of both LC3 I and LC3 II proteins was observed. The elevated level of LC3 II confirms the activation of autophagy. Additionally, the rise in LC3 I suggests that PPI may upregulate LC3 expression at the transcriptional level, potentially through the stabilization of LC3 mRNA, thereby enhancing the availability of LC3 I for lipidation.

Emerging research suggests that autophagy also plays a regulatory role in the process of cancer metastasis. EMT is a crucial mechanism facilitating cancer spreading and dissemination by enabling epithelial-polarized cells to lose their polarity and cell–cell adhesion, while acquiring mesenchymal characteristics associated with motility and invasiveness. EMT transcription factors (EMT-TFs) serve as the principal inducers of tumor metastasis, and their overexpression is correlated with an unfavorable prognosis in cancer [48]. The treatment of wild-type MMH hepatocytes with TGFB1 leads to the suppression of autophagy, while starvation-induced autophagy inhibits TGFB1-mediated EMT. Additionally, SNAI1 undergoes degradation through SQSTM1-dependent autophagy, thereby demonstrating a direct mechanism by which autophagy regulates EMT [49]. The stimulation of autophagy has been demonstrated to result in the downregulation of Snail and Slug, thereby triggering a molecular transition from a mesenchymal phenotype to an epithelial-like state in GBM cellular models. Consequently, this inhibits the invasive capability of GBM cells [50]. Our findings demonstrated that PPI effectively downregulated the expression of ZEB1, Snail, Slug, and Twist at both the RNA and protein levels, thereby highlighting its inhibitory impact on tumor EMT. Conversely, autophagy primarily suppresses EMT-induced tumor metastasis. Moreover, based on evidence in the literature, it has been suggested that PPI may reverse the EMT process through autophagy. To further support this notion, we conducted CHX chase experiments that led to the upregulated expression of p62 and LC3 II, which revealed that PPI promoted the accelerated consumption and degradation of β-catenin by inducing autophagy.

In summary, our results demonstrate that PPI effectively inhibits the growth and metastasis of cervical cancer in vitro and in vivo by modulating the β-catenin pathway. Specifically, PPI directly binds to the Ser552 residue of β-catenin, leading to a reduction in its expression through autophagy-mediated degradation. This subsequently suppresses TCF/LEF transcriptional activity and downregulates the expression of EMT-TFs, thereby repressing tumor invasion and metastasis.

While our SPR and CETSA experiments have proven the direct binding of PPI to the β-catenin protein, this interaction has also been confirmed through molecular docking analysis. Unfortunately, co-crystallization experiments have not yet been conducted, leaving the complex modes of interaction between compounds and target proteins unexplored and undefined. Furthermore, we have uncovered that PPI induces the degradation of the β-catenin protein through the autophagy–lysosomal pathway; however, further investigation is required to elucidate its underlying mode and mechanism of action.

Collectively, this study represents the pioneering evidence of a direct interaction between PPI and β-catenin protein, elucidating the inhibitory effect of PPI on CC metastasis through its direct modulation of the β-catenin signaling pathway, as well as the promotion of β-catenin degradation via autophagy. These findings hold immense scientific significance and possess substantial potential for novel drug development.

## 4. Materials and Methods

### 4.1. Chemicals and Reagents

Bafilomycin A1 (HY-100558, PubChem CID: 6436223, ≥98% purity), cisplatin (HY-17394, CID: 5702198, ≥98% purity), cycloheximide (HY-12320, CID: 6197, 99.82% purity), Polyphyllin I (HY-N0047, CID: 11018329, ≥98% purity), and MSAB (HY-120697, CID: 1159052, ≥98% purity) were purchased from MedChemExpress (MCE, Shanghai, China); MG132 (M8699, Sigma- Aldrich, Missouri, USA, CID: 462382) was dissolved in DMSO (543900, Sigma- Aldrich, Missouri, USA, CID: 679) and was stored at –80 °C.

Antibodies against β-catenin (1:1000, 8480T, Cell Signaling Technology, Danvers, MA, USA), E-cadherin (1:1000, PB9561, Boster, Wuhan, China), VE-cadherin (1:1000, AFRM9373, AiFang Biological, Changsha, China),Vimentin (1:1000, AFRM9324, AiFang Biological, Changsha, China), ZEB1 (1:1000, AF8388, Beyotime Biotechnology, Shanghai, China), Slug (1:1000, 9585T, Cell Signaling Technology, Danvers, MA, USA), phospho-β-catenin-ser552 (1:1000, 28778-1-AP, Proteintech, Wuhan, China), Snail (1:1000, AF8013, Beyotime Biotechnology, Shanghai, China), Twist (1:1000, AF8274, Beyotime Biotechnology, Shanghai, China), P62/SOSTM1 (1:1000, BM4385, Boster, Wuhan, China), LC3B (1:200, PTM-6384, PTMab, Hangzhou, China), GAPDH (1:1000, GB11002, Servicebio, Wuhan, China), β-actin (1:1000, GB15003, Servicebio, Wuhan, China), and β-tubulin (1:1000, AF2835, Beyotime Biotechnology, Shanghai, China) were obtained. All primary antibodies were raised in rabbits, except for β-tubulin, which was derived from mice.

### 4.2. Animals

The experiment involved the intraperitoneal (i.p.) injection of 8 × 10^6^ luciferase-labeled HO-8910PM cells into female BALB/c nude 6-week-old mice, resulting in the development of tumor nodules that closely resemble the gross pathology and dissemination pattern observed in human metastatic cervical cancer. The IVIS Lumina Bioluminescence Imaging System (IVIS; PerkinElmer, Hopkinton, MA, USA) was utilized for optical imaging to appraise the luciferase expression in the mouse model. Once the level of bioluminescence reached 1 × 10^7^, tumor-bearing mice were stochastically consigned to one of four groups—the control group, 4 mg/kg PPI group, 8 mg/kg PPI group, and 3 mg/kg cisplatin (cDDP) group (6 mice per group). PPI was administered via i.p. injection at the indicated dose on a daily basis, while cisplatin was administered via i.p. injection [51] at the indicated dose once every three days. All treatments were conducted for a duration of 3 weeks. The animal experimentation and usage protocols underwent review and approval by the Shanghai University of Traditional Chinese Medicine; animal care was in compliance with the Institutional Guidelines (approval number: PZSHUTCM2311070002).

### 4.3. Cell Lines and Culture

The HO-8910PM, SiHa, and C33A cells were obtained from QuiCell Biotechnology (Shanghai, China) and were authenticated using short tandem repeat (STR) profiling. The HO-8910PM cell line was cultured in RPMI 1640 medium with 10% FBS, 100 U/mL penicillin, and 100 mg/mL streptomycin at 37 °C in 5% CO_2_. The SiHa and C33A cell lines were cultured in MEM medium with 10% FBS, 100 U/mL penicillin, and 100 mg/mL streptomycin at 37 °C in 5% CO_2_.

### 4.4. Cell Viability Assay

Cell viability was measured via the MTT assay [52]. In short, 5000 cells were seeded per well in the 96-well plate. Following overnight incubation, the cells were subjected to a range of concentrations of PPI or to a control for 48 h. Subsequently, the cell viability was evaluated by measuring absorbance at 570 nm to assess formazan crystal formation. The experiment was conducted at least six times under the exact same conditions.

### 4.5. Wound Healing Assay

The HO-8910PM, SiHa, and C33A cells (6 × 10^5^ cells/well) were cultured in a 6-well plate and were left to adhere for 24 h. To maintain optimal cell growth, a linear scratch was made across the cell monolayer using a 200 µL pipette tip when the fusion degree of the monolayer exceeded 80% [53]. After three washes with phosphate-buffered saline (PBS), PPI at concentrations of 0, 0.5, and 1 µM without FBS was added to each plate, which was subsequently incubated at 37 °C in a 5% CO_2_ environment for 24 h. Microscopic photographs were taken at the start (0 h) and after 24 h following wound scratching (N-SIM, Nikon, Tokyo, Japan). The migration distance of cells was captured using a light microscope at 0 and 24 h for subsequent calculations. The migration rates of cells were determined using ImageJ 1.52a software (NIH, Bethesda, MD, USA).

### 4.6. Cell Migration and Invasion Assays

The cell migration assay [54] was performed by seeding cells onto transwell permeable support inserts that contain an 8 μm microporous membrane (from Corning Costar, Tewksbury, MA, USA) within 24-well plates. A total of 200 μL of HO-8910PM, SiHa, and C33A cell suspension (5 × 10^5^ cells/mL) in serum-free medium with 0, 0.5, and 1 μM PPI was added to the upper chambers, and 600 μL of culture medium containing 10% FBS was used as a chemoattractant in the lower compartment. After incubation for 24 h, the non-invasive cells were carefully removed, and the lower-surface migrated cells were fixed and stained using crystal violet or eosin. Subsequently, the stained cells were quantified by being counted in 8 randomly selected fields (100× magnification) per well. The transwell in the invasion assay was pre-smeared with Matrigel (Becton Dickinson Labware, Franklin Lakes, NJ, USA). Each experiment was repeated at least six times.

### 4.7. Quantitative Real Time PCR (qRT–PCR)

The HO-8910PM cells were treated with 0, 0.5, and 1 μM PPI for 6 h to detect the levels of β-catenin and for 24 h to detect levels of ZEB1, Twist, Slug, and Snail. The total RNA was extracted from HO-8910PM cells with TRIzol [55] (Vazyme, Nanjing, China). The RNA concentration was determined using the DeNovix DS-11 Lite Spectrophotometer (Delaware, USA). Subsequently, cDNA synthesis was performed on 1 μg of RNA using the HiScript^®^ II Q RTSuperMix for qPCR reagent Kit (Vazyme, Nanjing, China) according to the manufacturer’s instructions. The qPCR was run using ChamQ Universal SYBR qPCR Master Mix (Vazyme, Nanjing, China) on the QuantStudio 6 Flex system (Thermo Fisher Scientific, Massachusetts, USA). The thermocycling conditions employed were as follows: an initial denaturation at 95 °C for 30 s, followed by 40 cycles of denaturation at 95 °C for 5 s and annealing/extension at 60 °C for 30 s. The protocol concluded with a melting curve analysis ranging from 65 °C to 95 °C with 0.5 °C increments. The qPCR primers were constructed at Sangon Biotech, Shanghai. The expression values of the β-catenin, ZEB1, Twist, Slug, and Snail genes were standardized to the corresponding expression level of the internal control gene, Gapdh. The primer sequences are presented in Table 1.

### 4.8. Surface Plasmon Resonance (SPR)

The β-catenin protein (11279-H20B, Sinobiological, Beijing, China) was attached to the CM5 SPR sensor chip (Cytiva, MA, USA) through amine coupling. SPR experiments [56] were run on a BIAcore T200 (Cytiva, Massachusetts, USA). PPI was diluted in a serial manner to various concentrations using HBS-EP + buffer and was subsequently passed through the chip. The K_D_ values were determined utilizing the kinetics/affinity model in the BIAcore T200 analysis software (serial number: 4Y7×23–1242). The analysis was performed at a temperature of 25 °C, with 120 s of association time and a flow rate of 20 μL/min.

### 4.9. Cellular Thermal Shift Assay (CETSA)

The HO-8910PM cells were evenly distributed in two Petri dishes with a diameter of 10 cm. As described by Martinez Molina D. et al. [57], once cell fusion reached 80–90%, the cells were washed with trypsin and PBS and resuspended in 450 μL of PBS containing freshly added protease inhibitors. The cells were lysed through three cycles of rapid freezing in liquid nitrogen (1 min), followed by thawing in water at room temperature (1 min). Subsequently, the resulting cell lysates were centrifuged at 20,000× *g* for 20 min at 4 °C and were equally distributed between five tubes. The centrifugation supernatant was transferred to new 1.5 mL EP tubes for BCA measurement and was incubated with 5 μM PPI or the same volume of DMSO, respectively, at 4 °C for 3 h. Subsequently, the cell lysates in each tube were heated at the given temperatures for 3 min before being held at room temperature for another 3 min. Finally, the soluble fractions were retrieved for Western blot analysis.

### 4.10. Molecular Modeling

The flexible docking procedure integrated in Discovery Studio 2021 was applied to investigate the binding mode between PPI and β-catenin (PDB id: 1QZ7). The structure of the ligand was obtained from PubChem (PubChem CID: 11018329). The protein and the small molecule were prepared according to the procedures for preparing proteins and ligands with default parameters, respectively. The CHARMm force field was used to minimize both the protein and the ligand.

In this study, the reported small molecular binding pocket [58] was also defined as the binding site. The selected residue was set as Ser552. The conformation method in Generate Ligand Conformations was chosen as being the most appropriate. All other parameters were kept as default.

### 4.11. TOPFlash Reporter Gene Assay

The TOPFlash assay was performed in a 96-well plate. For each well, the cells were transfected with 0.22 μg of TOPFlash using Lipofectamine 3000. After treatment with 0, 0.5, and 1 μM PPI for 24 h, the luciferase activity was measured using the Dual Luciferase Reporter Assay Kit (DL101, Vazyme) following a previously established protocol [59]. The TOPFlash activity was standardized based on the Renilla luciferase signals.

### 4.12. CHX Chase Assay

The HO-8910PM cells were treated with cycloheximide (MedChemExpress, Shanghai, China), and the cell protein samples were collected after 0, 4, and 8 h of 100 µg/mL cycloheximide treatment. The protein expression levels were detected by Western blot analysis to determine protein stability [60].

### 4.13. Western Blot Analysis

The cells were lysed, and the proteins were extracted with RIPA Lysis Buffer (Beyotime, Shanghai, China). Protein concentrations were measured using a BCA kit from the same manufacturer. The protein samples were subsequently subjected to Western blot analysis according to the previously described protocol [61]. Briefly, they were incubated with the primary antibody overnight. The secondary antibodies (HRP-conjugated goat anti-mouse and goat anti-rabbit, Beyotime, Shanghai, China) were incubated at room temperature for 2 h. Meilunbio^®^ fg Super Sensitive ECL Luminescence Reagent (Dalian Meilun Biotechnology, Dalian, China) was used to detect protein signals on the Azure Biosystem (Azure c600, Azure Biosystem™, Dublin, CA, USA).

### 4.14. Immunofluorescent (IF) Staining Analysis

The HO-8910PM cells in the logarithmic growth phase were digested and passaged for cell crawling. Upon adherence, they were cultured in media supplemented with 0, 0.5, and 1 μM PPI for 24 h. These cells were then fixed with 4% paraformaldehyde at room temperature for 15 min and treated with 0.5% TritonX-100 (Greagent, Shanghai, China)for 10 min. After rinsing with PBS, the crawling sections were blocked with 5% goat serum at room temperature for 30 min. The primary antibodies were incubated overnight at 4 °C. Subsequently, after another PBS rinse, the sections were incubated with the secondary antibodies (diluted 1:100) in the dark at room temperature for 1 h. Nuclei were stained with DAPI (Beyotime Biotechnology, Shanghai, China), which was diluted at a 1:100 ratio, in the dark at room temperature for 10 min. Finally, the sections were carefully removed from the 12-well plate, and anti-fluorescence quenching sealing solution was added dropwise to the cell-attached surface. This was followed by observation and photography using a fluorescence microscope (Nikon, N-SIM, Japan) [62].

Tumor tissues [63] were fixed in 4% paraformaldehyde for 24 h, embedded in paraffin, and sectioned at 4 μm thickness. Paraffin-embedded sections underwent dewaxing and rehydration through sequential immersion in xylene (two times for 10 min each) and gradient alcohol solutions (100%, 95%, 85%, and 70% for 5 min each), before being washed with PBS three times for 5 min each. Antigen retrieval was carried out by treating samples with citrate buffer (pH 6.0) or EDTA buffer (pH 9.0) at 95–100 °C for 30 min in a pressure cooker or water bath, followed by natural cooling and three washes with PBS. Sections were then blocked with 5% goat serum in PBS at room temperature for 1 h, before being incubated with appropriately diluted primary antibodies overnight at 4 °C. After washing with PBS, fluorescently labeled secondary antibodies (Alexa Fluor 488/594, diluted at 1:500) were applied for 1 h at room temperature in the dark. Nuclei were counterstained with DAPI (1 μg/mL) for 5 min, and slides were mounted with anti-fluorescence quenching mounting medium, avoiding air bubbles, and images were subsequently captured using a fluorescence microscope (Nikon, N-SIM, Japan).

### 4.15. Hematoxylin–Eosin Staining (HE)

First, the tissue specimens were fixed with 4% paraformaldehyde (PFA) before being embedded in paraffin. The paraffin-embedded blocks were sectioned into 5 µm slices. Next, the slides underwent a meticulous deparaffinization procedure in xylene. This was followed by a systematic rehydration process through a series of graded ethanol solutions. The dehydration process utilizing graded ethanol solutions was carried out as follows: samples were initially immersed in 70% ethanol for 2 min, followed by transfer to 80% ethanol for an additional 2 min, and then treated with 95% ethanol for another 2 min period. Finally, two consecutive dehydration steps were performed using absolute (100%) ethanol, each lasting 2 min. Thereafter, routine staining was performed according to the hematoxylin–eosin staining protocol [64]. Lastly, the stained sections were imaged using fluorescence microscope systems (Nikon, Ti2, Japan), allowing for the precise visualization and documentation of the tissue structures.

### 4.16. Statistical Analysis

The data were examined using GraphPad Prism 8, and the results are presented as mean ± standard error of the mean (SEM). One-way or two-way ANOVA was conducted for three or more groups. The results were taken as significant when *p* < 0.05.

## 5. Conclusions

In summary, we showcased the potent inhibitory effects of PPI on the growth and metastasis of CC cells in both in vitro and in vivo settings through its direct modulation of the Wnt/β-catenin signaling pathway, as well as the promotion of β-catenin degradation via autophagy. Our findings demonstate that PPI has promising potential as a targeted therapeutic agent for treating cervical cancer metastasis.

## Figures and Tables

**Table 1:** Primer sequences.

Primer	Sequence
β-catenin forward (human)	5′-AAAGCGGCTGTTAGTCACTGG-3′
β-catenin reverse (human)	5′-CGAGTCATTGCATACTGTCCAT-3′
Snail forward (human)	5′-CTGGGTGCCCTCAAGATGCA-3′
Snail reverse (human)	5′-CCGGACATGGCCTTGTAGCA-3′
Slug forward (human)	5′-GTTTCATCCAGGATCGAGCAG-3′
Slug reverse (human)	5′-CATCTTCTTCCAGATGGTGA-3′
ZEB1 forward (human)	5′-ACTGTTTGTAGCGACTGGATT-3′
ZEB1 reverse (human)	5′-TAAAGTGGCGGTAGATGGTA-3′
Twist forward (human)	5′-CGGCCTAGCGAGTGGTTCTT-3′
Twist reverse (human)	5′-AGGAAAGAGCGCGGCATAGT-3′
Gapdh forward (human)	5′-CAGGAGGCATTGCTGATGAT-3′
Gapdh reverse (human)	5′-GAAGGCTGGGGCTCATTT-3′

## Data Availability

Data will be made available on request.
